# Beyond awareness: the knowledge–attitude–practice disconnect in e-waste management in an informal settlement of Delhi

**DOI:** 10.3389/fpubh.2026.1935156

**Published:** 2026-09-23

**Authors:** Shivani Rao, Kinshuk Gupta, Mongjam Meghachandra Singh, Nandini Sharma

**Affiliations:** 1 Department of Community Medicine, Maulana Azad Medical College, New Delhi, India; 2 Independent Researcher, New Delhi, India

**Keywords:** electronic waste, environmental health, e-waste management, informal settlement, knowledge-attitude-practice (KAP), India

## Abstract

**Background:**

E-waste is among the fastest-growing waste streams globally, with India being a major contributor. Safe e-waste management remains suboptimal, particularly among vulnerable urban populations, where limited awareness and unsafe disposal practices may increase environmental and health risks. This study assessed knowledge, attitudes, and practices (KAP) related to e-waste management among residents of an informal settlement in Delhi.

**Methods:**

A community-based cross-sectional study was conducted among 425 adults selected through simple random sampling. KAP was assessed using a pretested, semi-structured questionnaire. The total score obtained by each participant was expressed as a percentage of the maximum possible score. Participants achieving ≥50% of the maximum score were categorized as having good/adequate KAP. Firth penalized logistic regression was used to identify factors associated with adequate knowledge, positive attitudes, and good practices. Spearman’s rank correlation was used to assess correlations among KAP scores, and relationships were further examined using a hypothesized structural path model with indirect effects estimated from 5,000 bootstrap resamples and bias-corrected 95% confidence intervals.

**Results:**

Adequate knowledge was present in 17.18% of participants, positive attitudes in 44.47%, and good e-waste management practices in only 11.29%. Higher educational attainment was associated with adequate knowledge and a positive attitude, while younger participants, paid employment and ownership of >7 electronic gadgets were associated with higher odds of adequate knowledge and good practice. Knowledge, attitude, and practice scores were positively correlated (Spearman’s *ρ* = 0.253–0.481; all *p* < 0.001). In the path model, knowledge was positively associated with attitude and practice, while attitude was also positively associated with practice; however, the indirect pathway through attitude was not statistically significant (*b* = 0.013; bootstrap 95% CI: −0.001 to 0.026; *p* = 0.063).

**Conclusion:**

A substantial knowledge–attitude–practice gap in e-waste management was observed: despite modest knowledge and comparatively more favorable attitudes, safe disposal practices remained exceptionally low, and attitude did not significantly mediate the knowledge–practice association, with knowledge instead retaining a direct association with practice. Community-level e-waste interventions should strengthen awareness of health risks and formal disposal pathways while improving access to authorized collection and recycling services, with their effectiveness evaluated through future longitudinal and intervention studies.

## 1 Introduction

Rapid urbanization, accelerating technological innovation, and an increasing dependence on electronic devices coupled with their increased obsolescence have led to a dramatic rise in electronic waste (e-waste), making it one of the fastest-growing streams of municipal solid waste worldwide. Electronic waste, also known as e-waste or Waste Electrical and Electronic Equipment (WEEE), refers to discarded, surplus, obsolete, or broken electrical and electronic devices (1, 2).

According to data reported to the Central Pollution Control Board (CPCB) under the E-Waste (Management) Rules, national e-waste generation in India was estimated at approximately 1.40 million tonnes (13,97,955.59 MT) in year 2024–25, an increase of nearly 11% over FY 2023–24. However, only 70.7% of this quantity was formally collected, dismantled, and recycled or disposed of through registered channels in FY 2024–25, up from 61.9% in FY 2023–24, indicating that almost a third of generated e-waste is still not captured by the formal system (3, 4). Delhi ranks among the largest generators of e-waste in the country.

Improper e-waste disposal practices, including open burning, informal recycling, and landfill dumping, pose substantial environmental and health risks. Recent literature implicates e-waste in contributing to soil degradation, air pollution, water pollution and global warming. Heavy metals present in e-waste can leach into the soil, percolate into groundwater, and disperse through air as particulate matter. These contaminants do not remain confined to ecological compartments; instead, they move through food chains and water systems, bioaccumulating in plants, animals, and ultimately humans.

95% of e-waste is collected, handled, and managed by informal providers who are routinely exposed to harmful elements such as lead, mercury, cadmium, arsenic, selenium, and glass, thereby predisposing them to dermal, neurological, respiratory, and reproductive hazards (5). Many of these workers operate under precarious socioeconomic conditions and face heightened occupational risks due to limited awareness, inadequate training, lack of personal protective equipment, and insufficient recycling facilities and infrastructure (6). A report by the Associated Chambers of Commerce and Industry of India (ASSOCHAM) revealed that almost 80% of the workforce engaged in e-waste collection experiences some respiratory conditions (6). Populations living near dumping sites are more exposed to gasses, fumes, and radioactive substances, which can have deleterious long-term impacts including teratogenicity, weakened immunity, and impairment of renal and brain function (7–10).

Concurrently, e-waste represents a paradoxical resource reservoir within urban settings. Often termed “urban mining,” the recovery of precious and rare earth metals from discarded electronics offers an opportunity to reduce dependence on virgin mining, conserve natural resources, and mitigate environmental harm (11). However, when such recovery is inefficient or informal, not only are valuable materials lost, but hazardous extraction practices may further intensify environmental contamination and human exposure.

The Government of India notified the E-Waste (Management) Rules, 2022, which came into force on 1 April 2023 and superseded both the E-Waste (Management) Rules, 2016 and the E-Waste (Management) Amendment Rules, 2018, introducing a certificate-based Extended Producer Responsibility (EPR) trading mechanism and revised registration and reporting obligations for producers, recyclers, and refurbishers (12). However, the effectiveness of such policy instruments depends substantially on the knowledge, attitudes, and practices (KAP) of individuals within the community who generate and dispose of e-waste. Therefore, the present study was conducted to assess the knowledge, attitudes, and practices related to e-waste management among residents of an informal settlement in Gokalpuri.

Conceptually, the knowledge–attitude–practice (KAP) framework used in this study assumes that accurate information (knowledge) shapes evaluative beliefs (attitude), which in turn influence the adoption of protective or pro-environmental behaviors (practice). This pathway is not automatic as individuals may possess adequate knowledge without developing favorable attitudes, and favorable attitudes may not be enacted as safe practices when structural, economic, or infrastructural barriers intervene. Building on this framework, we have pre-specified three hypotheses for testing: (H1) knowledge regarding e-waste management is positively associated with attitude toward e-waste management; (H2) attitude is positively associated with practice; and (H3) attitude statistically mediates the association between knowledge and practice.

## 2 Materials and methods

### 2.1 Study setting

The total study area consisted of Ganga Vihar, Gokalpuri, and Gokalpur Village, comprising 9,597 households with a total population of 54,614 people. Ganga Vihar is officially listed by the Delhi Development Authority (DDA) as an unauthoried colony (Reg. No. 1046), adjoining Gokulpur Village. Gokalpuri and its constituent areas were classified as an informal settlement on the basis of their unplanned layout, absence of a sanctioned development plan, and informal or semi-formal arrangements for land tenure and municipal service provision, consistent with internationally used operational criteria for informal settlements (13).

### 2.2 Study population

Participants were eligible for inclusion if they were aged 18 years or older, resided in Gokalpuri for at least 6 months at the time of the study and expressed willingness to participate. Individuals unable to comprehend or respond to the interview questions because of severe cognitive impairment, speech/hearing disability, or terminal illness were excluded from the study.

The study was conducted for a period of 2 months starting from February 2026 to March 2026. Assuming a prevalence of adequate disposal practices of 47.5% from Bandyopadhyay A et al., with 95% confidence (*Z* = 1.96) and 5% absolute precision (*d* = 0.05), the calculated sample size (*n* = *Z*^2^*pq*/*d*^2^) is 383, which after adding 10% for non-response is rounded to 425 participants (14).

A simple random sampling with equal allocation across three localities was used. A line-listing of households was obtained from the Accredited Social Health Activist (ASHA) and Auxiliary Nurse Midwife (ANM) records (13). The study area comprised three geographic localities within the Urban Health Centre field practice area, namely Ganga Vihar, Gokalpuri, and Gokalpuri Village, which served as the sampling strata. Within each locality, households were selected using a computer-generated random number sequence applied to the household line-listing. One adult respondent was interviewed in each selected household. The head of the household was approached first, and if unavailable, the next eligible household member present at the time of the visit was interviewed instead. Where more than one eligible member was present, a Kish grid was used to randomly select one respondent and to avoid clustering of participants from the same household.

A total of 450 households were approached for participation. Of these, 425 eligible participants consented and completed the interview, yielding a response rate of 94.4%. Seventeen households refused participation, while eight households remained unavailable despite three visits and were considered non-respondents.

### 2.3 Data collection

The data collection was carried out through face-to-face interviews conducted by the investigators at participants’ homes. Prior to the interviews, researchers introduced themselves, explained the study’s objectives and procedures in simple and understandable language, and obtained written informed consent. For participants who were illiterate, the consent form was read aloud in the presence of a witness, and verbal consent was obtained and documented with a fingerprint in place of a signature. Data were collected through face-to-face interviews, with questions read aloud and responses recorded by the trained investigator for participants with limited literacy.

### 2.4 Study tools

As no standardized questionnaire assessing knowledge, attitude, and practices regarding e-waste management in the Indian setting was available, a semi-structured questionnaire was developed after an extensive review of the literature and previously published KAP studies. The draft questionnaire was reviewed by experts in Community Medicine and Environmental Health for its relevance, clarity, comprehensiveness, and suitability for the local context. Based on their suggestions, modifications were made to improve the wording and sequencing of items. It was translated into Hindi and independently back-translated into English to ensure semantic and conceptual equivalence.

The study instrument comprised a structured questionnaire with sections on sociodemographic characteristics and knowledge, attitude, and practices (KAP) related to e-waste management. Sociodemographic variables included age, gender, religion, marital status, education, occupation, type of family, nature of residence, and socioeconomic status (B. G. Prasad Classification) (15), along with details on the presence and number of discarded, replaced, or non-functional electronic items in the household. Additional information was collected on the frequency and speed of replacement of electronic devices, reasons for replacement (such as malfunction, technological obsolescence, or consumer preference), and sources of information regarding e-waste and its management.

Knowledge was assessed using 14 dichotomous items (yes/no), covering multiple domains including awareness of the concept of e-waste, identification of electronic waste sources, recognition of official symbols, and understanding of its health and environmental impacts. Attitude was assessed using 12 items measured on a combination of Likert and binary scales, capturing perceptions of responsibility, risk, and willingness to engage in appropriate management practices. All items were standardized to a common binary scoring metric before being summed to derive the composite percentage score (16). Strongly agree and agree were scored as 1, while neutral, disagree, and strongly disagree were scored as 0 for Likert Scale while Yes was scored as 1 and No as 0 for binary items. Practices were assessed using 4 dichotomous items evaluating actual behaviors related to e-waste disposal, segregation and use of protective measures.

The questionnaire was subsequently pretested among approximately 10% of the estimated sample at the Rural Health Training Centre (RHTC), Barwala, which was not included in the final study area. The pretest assessed the clarity, comprehensibility, and feasibility of administration, and minor revisions were incorporated before final data collection. For each KAP domain, the total item score was divided by the maximum possible score and multiplied by 100 to obtain a percentage score. A cut-off of 50% was then used to dichotomise the scores, with scores ≥50% classified as adequate knowledge, positive attitude, or good practice, and scores <50% classified as inadequate knowledge, negative attitude, or poor practice.

Internal consistency was assessed using Cronbach’s alpha. The knowledge domain demonstrated good reliability (Cronbach’s *α* = 0.87), while the attitude domain showed acceptable reliability (Cronbach’s *α* = 0.71). The practice domain consisted of a limited number of conceptually distinct behavioral items; therefore, internal consistency was not considered an appropriate measure of reliability, and the items were retained based on expert opinion and their relevance to e-waste management practices.

### 2.5 Ethics approval

Institutional Ethics Committee permission was obtained before the start of the study (the Institutional Ethics Committee of Maulana Azad Medical College/IEC/2021/388 dated February 06, 2026). Our study was conducted according to the Declaration of Helsinki. Written informed consent was obtained from all participants prior to data collection. Participation was voluntary, and respondents were informed of their right to withdraw at any time without consequences. Confidentiality and privacy were strictly maintained. No personal identifiers were collected, and the anonymised dataset was stored securely, with day-to-day access restricted to the research team during the conduct of the study. Participants with unmet needs were referred to UHTC Gokalpuri for further management.

### 2.6 Data management and statistical analysis

Data were entered into KoboCollect (KoBoToolbox, Harvard Humanitarian Initiative, USA) and analyZed using STATA version 19 (StataCorp LLC, College Station, TX, USA). The percentage of correct responses was calculated by dividing the total number of correct answers by the total number of questions and multiplying the result by 100 for each domain (knowledge, attitude, and practice).

Continuous variables were assessed for normality using the Shapiro–Wilk test. Normally distributed variables were presented as mean ± standard deviation (SD), whereas non-normally distributed variables are reported as median with interquartile range (IQR). Categorical variables are presented as frequencies and percentages. There were no missing values for any sociodemographic, knowledge, attitude, or practice variable included in the analysis.

Associations between categorical independent variables and the dichotomised knowledge and attitude outcomes were assessed using the Chi-square test or Fisher’s exact test, as appropriate. Variables with a *p*-value <0.20 in the bivariate analysis, along with those considered epidemiologically relevant, were entered simultaneously into multivariable binary logistic regression models using a forced-entry (enter) approach to obtain mutually adjusted estimates. Given the low number of outcome events per predictor, Firth’s penalized logistic regression was used to reduce small-sample bias and quasi-separation in some cells to obtain more stable estimates. Results are presented as adjusted odds ratios (aORs) with 95% confidence intervals (CIs) and two-tailed *p*-values. Overall model significance was assessed using the Wald χ^2^ statistic.

A structural path model was estimated to test the hypothesis regarding the relationships among knowledge, attitude, and practice. All path coefficients are reported as unstandardised coefficients (b) in the metric of the composite percentage scores, with bias-corrected bootstrap confidence intervals derived from 5,000 resamples.

All statistical tests were two-tailed, and a *p* value <0.05 was considered statistically significant.

## 3 Results

### 3.1 Sociodemographic profile

A total of 425 participants were included in the analysis. The mean age was 42.07 years (SD = 13.30; range 18–75). The majority of the participants were females (62.59%), married (78.35%), and belonged to Hindu religion (96.00%) (Table 1). Graduates constituted the largest group (27.06%), and over half of respondents were homemakers (52.24%). Most participants resided in nuclear family (61.88%), owned a house (87.53%), and belonged to upper middle socioeconomic status under BG Prasad socioeconomic status (64.71%).

**Table 1:** Sociodemographic status of residents (*N* = 425).

Characteristics	*n* (%)
Age (in completed years)	
18–29	88 (20.71)
30–39	106 (24.94)
40–49	96 (22.59)
50–59	79 (18.59)
60–69	48 (11.29)
70 and above	8 (1.88)
Gender	
Female	266 (62.59)
Male	157 (36.94)
Transgender	2 (0.47)
Religion	
Hindu	408 (96.00)
Muslim	11 (2.59)
Others	6 (1.41)
Marital status	
Single	72 (16.94)
Married	333 (78.35)
Divorced/Separated/Widowed	20 (4.71)
Education level	
Illiterate	51 (12.00)
Primary school	63 (14.82)
Middle school	54 (12.71)
High school	53 (12.47)
Higher secondary school	76 (17.88)
Graduate	115 (27.06)
Professional/Postgraduate	13 (3.06)
Occupation	
Unemployed	47 (11.06)
Homemaker	222 (52.24)
Self-employed	17 (4.00)
Government job	9 (2.12)
Private job	97 (22.82)
Student	33 (7.76)
Type of family	
Nuclear	263 (61.88)
Three generation	124 (29.18)
Joint	38 (8.94)
Nature of residence	
Own house	372 (87.53)
Rented house	53 (12.47)
Socioeconomic status	
Upper class	66 (15.53)
Upper middle class	275 (64.71)
Middle class	79 (18.59)
Lower middle class	3 (0.71)
Lower class	2 (0.47)

### 3.2 Household digital access, mobile usage patterns, and information sources

The majority of households possessed a moderate to high number of electronic gadgets, with nearly half (47.06%) reporting 7–9 devices, while 36.24% had 4–6 devices (Table 2). Only a small proportion (3.29%) had three or fewer gadgets. A large majority of households (94.12%) reported no unused or non-working electronics at home. Most households (86.35%) had not replaced any electronic devices in the past year, while only 10.12% replaced one device.

**Table 2:** Household digital access, mobile usage patterns, and information sources among study participants (*N* = 425).

Characteristics	*n* (%)
Number of gadgets in household	
1–3	14 (3.29)
4–6	154 (36.24)
7–9	200 (47.06)
≥10	57 (13.41)
Unused electronics at home	
No	400 (94.12)
Yes	25 (5.88)
Devices replaced last year	
0	367 (86.35)
1	43 (10.12)
≥2	15 (3.53)
Number of mobile phones	
0–1	16 (3.76)
2–3	209 (49.18)
4–5	170 (40.00)
≥6	30 (7.06)
Mobile phone replacement interval (months)	
≤24	69 (16.24)
25–48	154 (36.24)
49–72	177 (41.65)
>72	25 (5.88)
Reasons for replacement of last mobile*	
Device old	184 (43.29)
Battery issues	116 (27.29)
Repair not possible	80 (18.82)
Other reasons	74 (17.41)
New features	50 (11.76)
Got stolen	30 (7.06)
Sources of information of e-waste*	
No information	356 (83.76)
Internet/social media	42 (9.88)
School/colleges	32 (7.53)
Friends/relatives	13 (3.06)
User manuals	13 (3.06)
Mass media	11 (2.59)
Government campaigns	6 (1.41)
Newspaper	4 (0.94)
TV	3 (0.71)
Radio	2 (0.47)

*N* = 425. *Multiple options correct.

The mean number of mobile phones per household was 3.41 ± 1.42. Most households had multiple devices, with 49.18% reporting 2–3 phones. Most participants replaced their mobile phones after 2–6 years, with 41.65% reporting replacement after 49–72 months and 36.24% after 25–48 months, while only 5.88% reported replacing their phones after more than 72 months. Among reasons for mobile replacement, the most common reason was the device becoming old (43.29%), followed by battery issues (27.29%) and repair not being possible (18.82%). Replacement due to new features (11.76%) or theft (7.06%) was less common.

Awareness regarding electronics-related information sources was notably low. A substantial proportion of participants (83.76%) reported no access to any information source. Among those with exposure, internet and social media were the most common sources (9.88%), followed by schools/colleges (7.53%). Traditional media such as television, radio, and newspapers contributed minimally.

### 3.3 Knowledge, attitude and practice regarding e-waste management

Among the 425 participants, 73 (17.18%) had adequate knowledge, 189 (44.47%) had a positive attitude, and 48 (11.29%) reported good practices, based on the 50% score threshold.

#### 3.3.1 Knowledge

Knowledge was particularly limited regarding the basic concept, health hazards, and formal management of e-waste. Only 20.47% had heard of e-waste, while awareness of authorized recyclers and formal take-back systems was 2.35 and 2.59%, respectively. In contrast, almost all participants recognized environmental contamination from improper disposal (96.00%) and the need for segregation (96.24%) (Supplementary Table S1).

#### 3.3.2 Attitude

Most participants recognized the importance of e-waste management (79.06%) and household responsibility (99.06%). However, endorsement of individual and manufacturer responsibility was lower (34.12 and 23.29%, respectively), as was support for authorized collection facilities (3.29%). Recognition of the need for greater public awareness (87.76%) and support for community education (84.47%) were comparatively high (Supplementary Table S2).

#### 3.3.3 Practice

Good practices were uncommon (11.29%). Although 47.29% reported keeping unusable electronic items separate from household waste, only 18.12% reported segregation before disposal. Formal disposal through authorized facilities (1.41%) and use of protective measures (0.71%) were rare (Supplementary Table S3).

### 3.4 Predictors of knowledge, attitude and practice

In the multivariable Firth logistic regression model, higher education (aOR = 11.32, 95% CI: 2.92–43.86; *p* < 0.001), paid employment (aOR = 2.73, 95% CI: 1.26–5.92; *p* = 0.011), and ownership of >7 gadgets (aOR = 4.89, 95% CI: 2.46–9.75; *p* < 0.001) were independently associated with higher odds of adequate knowledge. Participants aged ≥40 years had lower odds of adequate knowledge (aOR = 0.29, 95% CI: 0.12–0.68; *p* = 0.005), while married participants also had lower odds (aOR = 0.42, 95% CI: 0.20–0.87; *p* = 0.019). Sex and socioeconomic status were not independently associated with adequate knowledge. The model was statistically significant (Wald χ^2^(7) = 65.13, *p* < 0.001), and no evidence of problematic multicollinearity was observed (VIF range: 1.03–1.79; mean VIF = 1.43) (Table 3).

**Table 3:** Multivariable binary logistic regression: independent predictors of adequate knowledge regarding e-waste management among study participants (*N* = 425).

Variable	Category	Crude OR	95% CI	*p*-value	Adjusted OR	95% CI	*p*-value
Sex	Male/Other vs. Female	2.72	1.38–5.36	0.004*	1.53	0.72–3.26	0.269
Age group	≥40 years vs. < 40 years	0.15	0.06–0.36	<0.001*	0.29	0.12–0.68	0.005*
Marital status	Married vs. Not currently married	0.32	0.16–0.63	0.001*	0.42	0.20–0.87	0.019*
Educational status	Higher secondary and above vs. Up to secondary	31.84	6.15–165.00	<0.001*	11.32	2.92–43.86	<0.001*
Occupation	Paid employment vs. Not in paid employment	6.02	2.95–12.29	<0.001*	2.73	1.26–5.92	0.011*
Number of gadgets	>7 vs. ≤ 7	3.16	1.56–6.41	0.001*	4.89	2.46–9.75	<0.001*
Socioeconomic status	Upper SES vs. other SES categories	2.61	0.85–8.06	0.095†	1.03	0.40–2.60	0.958

Model statistics: *N* = 425; Wald χ^2^(7) = 65.13; *p* < 0.001; penalized log likelihood = −107.10.

Unadjusted ORs were obtained from univariable logistic regression models. Adjusted ORs were estimated from a single multivariable logistic regression model including all eight variables simultaneously. Reference categories: Gender = Female/Transgender; Age group = ≥40 years; Marital status = Married; Educational status = Up to secondary; Occupation = Not in paid employment; Number of gadgets = ≤7 gadgets; Mobile replacement cycle = > 2 years; Socioeconomic status = Lower SES. OR, Odds Ratio; 95% CI, 95% Confidence Interval. *p < 0.05; †*p* < 0.10.

For attitude, higher education was the only independent predictor of a positive attitude (aOR = 2.50, 95% CI: 1.49–4.21; *p* = 0.001). The model was statistically significant (Wald χ^2^ = 35.97, *p* < 0.001). No evidence of problematic multicollinearity was observed (VIF range: 1.01–1.79; mean VIF = 1.38) (Table 4).

**Table 4:** Multivariable binary logistic regression: independent predictors of positive attitude toward e-waste management among study participants (*N* = 425).

Variable	Category	Crude OR (95% CI)	*p*-value	Adjusted OR (95% CI)	*p*-value
Sex	Male/Other vs Female	1.71 (1.15–2.54)	0.008*	1.52 (0.91–2.55)	0.110
Age group	≥40 years vs <40 years	0.51 (0.34–0.74)	0.001*	0.91 (0.55–1.50)	0.701
Marital status	Married vs Not currently married	0.51 (0.32–0.81)	0.005*	0.73 (0.44–1.22)	0.235
Educational status	Higher secondary and above vs Up to secondary	3.13 (2.10–4.66)	<0.001*	2.50 (1.49–4.21)	0.001*
Occupation	Paid employment vs Not in paid employment	1.61 (1.05–2.44)	0.027*	0.86 (0.49–1.50)	0.588
Number of gadgets	>7 vs ≤7	1.68 (1.14–2.48)	0.009*	1.27 (0.84–1.93)	0.261

Model statistics: *N* = 425; Wald χ^2^(6) = 35.97; *p* < 0.001; penalized log likelihood = −261.48.

Crude ORs were obtained from univariable Firth penalized logistic regression models. Adjusted ORs were estimated using a multivariable Firth penalized logistic regression model including all variables shown in the table simultaneously. Reference categories were: sex = Female; age group = <40 years; marital status = Not currently married; educational status = Up to secondary; occupation = Not in paid employment; number of gadgets = ≤7. OR, odds ratio; CI, confidence interval. *p < 0.05. †*p* < 0.10.

For practice, participants aged ≥40 years had significantly lower odds of good practice (aOR = 0.20, 95% CI: 0.08–0.49; *p* < 0.001), whereas paid employment (aOR = 4.53, 95% CI: 1.97–10.39; *p* < 0.001), ownership of >7 gadgets (aOR = 3.95, 95% CI: 1.84–8.51; *p* < 0.001), and >3 mobile phones (aOR = 2.23, 95% CI: 1.09–4.57; *p* = 0.028) were independently associated with higher odds of good practice. Gender showed a borderline association (aOR = 2.26, 95% CI: 0.97–5.24; *p* = 0.057). The model was statistically significant (Wald χ^2^(7) = 54.74, *p* < 0.001). No evidence of problematic multicollinearity was observed, with VIFs ranging from 1.01 to 1.79 (Table 5).

**Table 5:** Multivariable binary logistic regression: independent predictors of good e-waste management practices among study participants (*N* = 425).

Variable	Category	Crude OR (95% CI)	*p*-value	Adjusted OR (95% CI)	*p*-value
Gender	Male/Other vs. Female	4.40 (2.32–8.34)	<0.001*	2.26 (0.97–5.24)	0.057†
Age group	≥40 years vs. < 40 years	0.19 (0.09–0.39)	<0.001*	0.20 (0.08–0.49)	<0.001*
Marital status	Married vs. Not currently married	0.50 (0.26–0.95)	0.035*	1.07 (0.48–2.39)	0.876
Educational status	Higher secondary and above vs. Up to secondary	7.26 (3.25–16.22)	<0.001*	1.48 (0.56–3.97)	0.431
Occupation	Paid employment vs. Not in paid employment	8.60 (4.40–16.82)	<0.001*	4.53 (1.97–10.39)	<0.001*
Number of gadgets	>7 vs. ≤ 7	4.88 (2.48–9.59)	<0.001*	3.95 (1.84–8.51)	<0.001*
Number of mobile phones	>3 vs. ≤ 3	1.66 (0.91–3.03)	0.101†	2.23 (1.09–4.57)	0.028*

Model statistics: *N* = 425; Wald χ^2^(7) = 54.74; *p* < 0.001; penalized log likelihood = −96.05.

Crude ORs were obtained from univariable Firth penalized logistic regression models. Adjusted ORs were estimated using a multivariable Firth penalized logistic regression model including all seven variables shown in the table simultaneously. Reference categories were: sex = Female; age group = <40 years; marital status = Not currently married; educational status = Up to secondary; occupation = Not in paid employment; number of gadgets = ≤7; number of mobile phones = ≤3. OR, odds ratio; CI, confidence interval. *p < 0.05; †*p* < 0.10.

### 3.5 Correlation among KAP domains

Knowledge, attitude, and practice scores were positively correlated (Spearman’s *ρ* = 0.253–0.481; all *p* < 0.001), with the strongest correlation between knowledge and attitude (*ρ* = 0.481), followed by knowledge and practice (*ρ* = 0.384) and attitude and practice (*ρ* = 0.253).

### 3.6 Structural path analysis

In the hypothesized path model, knowledge was positively associated with attitude (*b* = 0.273, *β* = 0.468; *p* < 0.001), and attitude was positively associated with practice (*b* = 0.047, *β* = 0.111; *p* = 0.022). Knowledge also remained directly associated with practice (*b* = 0.102, *β* = 0.410; *p* < 0.001). The indirect association between knowledge and practice through attitude was small and not statistically significant (*b* = 0.013; bootstrap 95% CI − 0.001 to 0.026; *p* = 0.063). Thus, the hypothesized model did not provide evidence of a statistically significant mediating effect of attitude.

### 3.7 Barriers and facilitators to safe e-waste management

Multiple barriers to appropriate e-waste disposal were identified. The most frequently reported barrier was lack of awareness (79.76%). Nearly one-third of participants (29.88%) indicated the absence of nearby collection facilities as a constraint. Preference for scrap dealers was reported by 16.47%, reflecting reliance on informal disposal channels. Other barriers were less commonly cited, including lack of time (5.88%), absence of financial incentives (4.94%), and perceived inconvenience of the disposal process (4.00%).

Several facilitating factors were also identified. Awareness campaigns were the most widely endorsed strategy (76.94%), followed by door-to-door pickup services (39.29%) and the availability of convenient collection sites (20.94%). Financial incentives were supported by 9.65% of respondents, whereas legal enforcement through fines was endorsed by only 1.18%.

## 4 Discussion

This community-based cross-sectional study revealed a substantial knowledge-attitude-practice gap regarding e-waste management among residents of Gokalpuri, with only 17.18% of participants demonstrating adequate knowledge and 11.29% reporting good disposal practices, despite 44.47% exhibiting a positive attitude toward e-waste management. Higher education associated with adequate knowledge and a positive attitude. Younger, employed and participants who owned >7 electronic gadgets at their homes had higher odds of both good knowledge and practice. Knowledge, attitude, and practice scores were positively correlated. The hypothesized indirect pathway from knowledge to practice through attitude was not statistically significant, while knowledge retained a significant direct association with practice.

Our findings of low knowledge and low practice, alongside comparatively more favorable but still largely inadequate attitudes, are broadly comparable to studies done in other low- and middle-income countries (17–23). A study conducted by Meem RA et al. in Bangladesh found that while all participants generated e-waste, only 26.5% had adequate knowledge regarding its disposal (18). Similarly, Kariwala et al. in Lucknow observed that only 37% of participants were aware of the health impacts of e-waste (24). In contrast, Bandyopadhyay et al. reported substantially higher levels of awareness, with 93.4% of participants recognizing the harmful effects of e-waste (14). The lower awareness observed in our study may reflect differences in educational attainment, socioeconomic conditions, and access to information among the study populations.

However, knowledge was not uniformly low across all domains. While almost all participants recognized that improper disposal of e-waste could contaminate the environment (96.00%) and that e-waste should be segregated from household waste (96.24%), awareness of health hazards was limited, with only 9.88% recognizing its potential adverse effects on human health. Awareness of formal management systems was particularly poor, with only 2.35% aware of authorized recyclers and 2.59% aware of formal collection or take-back systems. Similar gaps in awareness of health effects and formal management have been reported in India. Kariwala et al. found that only 37% of participants in Lucknow were aware of the adverse health effects of e-waste, while 87.3% were unaware of government guidelines (24).

Education emerged as an important determinant of both knowledge and attitudes. Participants with higher educational attainment demonstrated significantly better knowledge, underscoring the critical role of education in shaping environmental awareness, risk perception, and informed decision-making regarding e-waste management. Similar associations have been reported in India and other low- and middle-income settings, where higher educational attainment has consistently been associated with greater awareness of e-waste hazards, safer disposal practices, and more favorable environmental attitudes (25–27).

The attitude findings also revealed a marked asymmetry in perceived responsibility. While almost all participants (99.06%) endorsed household-level responsibility for safe e-waste disposal, far fewer recognized individual responsibility (34.12%) or manufacturer responsibility (23.29%), and support for authorized collection facilities was particularly low (3.29%). This diffusion of responsibility away from the individual and the formal system, despite widespread recognition that e-waste management is important for health and the environment (79.06%), is a pattern also described in other community-based e-waste KAP studies from South Asia and sub-Saharan Africa, where favorable general attitudes have not consistently translated into support for formal collection systems (17, 21, 22, 27).

The predictors of good practice differed from those of knowledge and attitude. Younger age, paid employment, and greater ownership of electronic gadgets and mobile phones were independently associated with better practice, whereas educational attainment was the main independent predictor of knowledge and attitude. The barriers reported by participants provide additional context for these findings, with lack of awareness of disposal mechanisms (79.76%) and absence of nearby collection facilities (29.88%) being the most frequently reported barriers. These findings suggest that appropriate e-waste management may depend not only on individual knowledge and attitudes but also on the availability and accessibility of formal disposal options. Consistent with this, awareness campaigns (76.94%) and door-to-door collection services (39.29%) were the most commonly endorsed facilitators, highlighting awareness and convenient access to disposal services as potential priorities for e-waste management programs in similar settings.

The high willingness of participants to attend community-based workshops on e-waste management offers an important opportunity for intervention, particularly because a large proportion of participants reported not receiving any prior information regarding e-waste. Strengthening community awareness of e-waste health risks and formal disposal pathways, together with improving access to authorized collection and recycling services, may help address the gaps identified in this study. Consumer awareness and disposal behavior remain central to the success of e-waste management interventions. Without active public participation and accessible disposal pathways, efforts aimed at safe recycling, pollution reduction, and resource recovery are unlikely to achieve their intended outcomes (28–30).

The present study provides useful insights into an emerging environmental and public health concern in India. Future studies should employ longitudinal and mixed-methods designs to better understand the pathways linking knowledge, attitudes, and e-waste management practices. Implementation studies evaluating educational programs and improvements in access to formal e-waste collection systems are also needed to identify effective strategies for promoting safe disposal behaviors.

This study has several limitations. The study was conducted in a single urban informal settlement, which may limit the generalisability of the findings to other populations and settings. Its cross-sectional design does not allow causal or temporal relationships between knowledge, attitudes, and practices to be established in the mediation analysis. Although the questionnaire underwent literature review, expert review, pretesting, translation and back-translation, and internal-consistency assessment, it was not formally validated for construct validity. Selection of available household members may have introduced selection bias, particularly over-representing women, homemakers, and older adults. Finally, self-reported practices may be affected by recall and social desirability bias.

## 5 Conclusion

This study found that e-waste management practices in an urban informal settlement of Delhi remained poor despite some awareness and relatively favorable attitudes among residents of the study area. Adequate knowledge was observed in only 17.18% of participants, compared with 44.47% with a positive attitude and 11.29% reporting good practices. Although knowledge, attitude, and practice were positively correlated, the hypothesized pathway through which knowledge influenced practice via attitude was not statistically significant. Community-level e-waste interventions should strengthen awareness of health risks and formal disposal pathways while improving access to authorized collection and recycling services, with their effectiveness evaluated through future longitudinal and intervention studies.

## Data Availability

The raw data supporting the conclusions of this article will be made available by the authors, without undue reservation.
