# Supplementary material for: Beyond awareness: the knowledge–attitude–practice disconnect in e-waste management in an informal settlement of Delhi
Source: Front Public Health. 2026 Sep 23;14:1935156. doi: 10.3389/fpubh.2026.1935156 (PMC13642671; doi:10.3389/fpubh.2026.1935156)
Supplement: Supplementary file 1 [file Supplementary_file_1.docx]

**Supplementary Table S1. Item-level knowledge regarding e-waste management among study participants (N=425)**

| **No.** | **Knowledge question** | **Response allowed** | **Response** | **n (%)** |
| --- | --- | --- | --- | --- |
| **A. Basic understanding of e-waste** | | | | |
| 1 | Have you heard of the term “e-waste” or “electronic waste”? | Yes/No | Yes/No | 87 (20.47) |
| 2 | Have you seen the symbol of “e-waste”? | Yes/No | Yes/No | 32 (7.53) |
| 3 | When an electrical or electronic product is no longer wanted or usable and is discarded, is it considered e-waste? | Yes/No | Yes/No | 119 (28.00) |
| **B. Health and environmental hazards** | | | | |
| 4 | Can e-waste contain substances that may be harmful to human health? | Yes/No | Yes/No | 42 (9.88) |
| 5 | Can e-waste contain toxic metals such as lead, mercury and cadmium? | Yes/No | Yes/No | 83 (19.53) |
| 6 | Can unsafe handling or dismantling of e-waste expose people to health risks? | Yes/No | Yes/No | 48 (11.29) |
| 7 | Can burning e-waste release harmful substances into the air? | Yes/No | Yes/No | 135 (31.76) |
| 8 | Can improper disposal of e-waste contaminate air, water and soil? | Yes/No | Yes/No | 408 (96.00) |
| **C. Recycling and safe disposal** | | | | |
| 9 | Can useful materials such as metals be recovered from e-waste through recycling? | Yes/No | Yes/No | 76 (17.88) |
| 10 | Should e-waste be separated from ordinary household waste before disposal? | Yes/No | Yes/No | 409 (96.24) |
| 11 | Should unwanted electronic items be taken to designated e-waste collection or recycling facilities? | Yes/No | Yes/No | 99 (23.29) |
| **D. Formal e-waste management** | | | | |
| 12 | Are there authorized or registered recyclers who can safely manage e-waste? | Yes/No | Yes/No | 10 (2.35) |
| 13 | Can consumers return unwanted electronic products through formal collection or take-back systems? | Yes/No | Yes/No | 11 (2.59) |

**Supplementary Table S2: Item-level attitudes towards e-waste management among study participants (N=425)**

| **S. No.** | **Attitude question** | **Response format** | **Positive attitude, n (%)** |
| --- | --- | --- | --- |
| **Perceived importance and responsibility** | | | |
| 1 | How strongly do you agree or disagree that proper management of e-waste is important for protecting human health and the environment? | 5-point Likert | 336 (79.06) |
| 2 | Do you believe that every household has a responsibility to manage its e-waste safely? | Yes/No | 421 (99.06) |
| 3 | Do you believe that recycling unwanted electronic products is beneficial for the community and environment? | Yes/No | 326 (76.71) |
| **Responsibility for e-waste management** | | | |
| 4 | How strongly do you agree or disagree that individuals should take responsibility for ensuring that their e-waste is disposed of safely? | 5-point Likert | 145 (34.12) |
| 5 | Do you believe that manufacturers should take responsibility for managing e-waste from their products? | Yes/No | 99 (23.29) |
| 6 | How strongly do you agree or disagree that local authorities should take an active role in e-waste management? | 5-point Likert | 47 (11.06) |
| **Formal e-waste management** | | | |
| 7 | Do you support the use of authorized collection and recycling facilities for e-waste? | Yes/No | 14 (3.29) |
| 8 | Do you believe that easily accessible e-waste collection facilities would make responsible disposal easier for households? | Yes/No | 34 (8.00) |
| 9 | Do you believe that community-based e-waste collection and recycling systems should be developed? | Yes/No | 73 (17.18) |
| **Community awareness and action** | | | |
| 10 | Do you believe that community participation can improve e-waste management? | Yes/No | 49 (11.53) |
| 11 | Do you believe that greater public awareness is needed to improve e-waste management? | Yes/No | 373 (87.76) |
| 12 | Do you support community education programmes on safe e-waste management? | Yes/No | 359 (84.47) |

**Supplementary Table S3: Item-level e-waste management practices among study participants (N=425)**

| **S. No.** | **Practice question** | **Response** | **n (%)** |
| --- | --- | --- | --- |
| **Household handling** | | | |
| 1 | When an electronic item is no longer usable, do you keep it separately from your other household waste? | Yes/No | 201 (47.29) |
| **Segregation** |  |  |  |
| 2 | Before disposing of an unwanted electronic item, do you separate it from your other household waste? | Yes/No | 77 (18.12) |
| **Formal disposal** | | | |
| 3 | Have you ever taken an unwanted electronic item to an authorized e-waste collection centre or registered recycler? | Yes/No | 6 (1.41) |
| **Safe handling** | | | |
| 4 | When handling discarded electronic items, do you use protective measures such as gloves to avoid direct contact with potentially harmful components? | Yes/No | 3 (0.71) |
